# RNA-Binding Proteins in Amyotrophic Lateral Sclerosis and Neurodegeneration

**DOI:** 10.1155/2012/432780

**Published:** 2012-04-19

**Authors:** Scott E. Ugras, James Shorter

**Affiliations:** ^1^Biochemistry and Molecular Biophysics Graduate Group, Perelman School of Medicine, University of Pennsylvania, Philadelphia, PA 19104, USA; ^2^Department of Biochemistry and Biophysics, Perelman School of Medicine, University of Pennsylvania, 805b Stellar-Chance Laboratories, 422 Curie Boulevard, Philadelphia, PA 19104, USA

## Abstract

Amyotrophic Lateral Sclerosis (ALS) is an adult onset neurodegenerative disease, which is universally fatal. While the causes of this devastating disease are poorly understood, recent advances have implicated RNA-binding proteins (RBPs) that contain predicted prion domains as a major culprit. Specifically, mutations in the RBPs TDP-43 and FUS can cause ALS. Cytoplasmic mislocalization and inclusion formation are common pathological features of TDP-43 and FUS proteinopathies. Though these RBPs share striking pathological and structural similarities, considerable evidence suggests that the ALS-linked mutations in TDP-43 and FUS can cause disease by disparate mechanisms. In a recent study, Couthouis et al. screened for protein candidates that were also involved in RNA processing, contained a predicted prion domain, shared other phenotypic similarities with TDP-43 and FUS, and identified TAF15 as a putative ALS gene. Subsequent sequencing of ALS patients successfully identified ALS-linked mutations in TAF15 that were largely absent in control populations. This study underscores the important role that perturbations in RNA metabolism might play in neurodegeneration, and it raises the possibility that future studies will identify other RBPs with critical roles in neurodegenerative disease.

## 1. Introduction

Amyotrophic Lateral Sclerosis (ALS) is an adult-onset neurodegenerative disease that is typically fatal within 2–5 years of disease onset. Loss of upper and lower motor neurons leads to paralysis and is usually the cause of death. Most cases are sporadic with no family history, but ~10% of cases are familial [1].

Efforts to understand the causes of ALS and methods to mitigate its pathogenesis have been severely limited by an inability to identify ALS genes. For the past two decades, most ALS research has focused on the ubiquitously expressed copper/zinc superoxide dismutase 1 (SOD1). Mutations in *SOD1* underlie ~20% of familial ALS (fALS) cases and ~1% of sporadic ALS (sALS) cases [1]. No consensus has emerged on the mechanism by which these SOD1 mutations lead to selective neuronal death in ALS. However, loss of SOD1 function does not cause motor dysfunction and ALS-linked SOD1 mutants can have wild-type enzyme activity [2]. Thus, a toxic gain of function of misfolded SOD1 likely underpins pathogenesis [2]. Recently, a number of genes involved in RNA processing have been identified that can cause ALS in select cases [3–7]. These findings have revolutionized our understanding of ALS and have simultaneously changed the genetic landscape of neurodegenerative disease.

## 2. TDP-43 Is an ALS Disease Protein

 The identification of a novel ALS disease protein in 2006 dramatically altered our understanding of this disease and brought to light the potential role RNA-binding proteins (RBPs) play in neurodegeneration. TDP-43 was found in cytoplasmic inclusions in diseased brain [3]. Shortly thereafter, many mutations in *TDP43 *were found in sALS and fALS cases, but not in control cases [6, 7]. This finding took the ALS community by surprise and caused many researchers to focus on TDP-43 and the role other RBPs might play in ALS.

TDP-43 is a highly conserved, ubiquitously expressed protein that has various roles in RNA metabolism [8, 9]. TDP-43 contains two RNA Recognition Motifs (RRMs) and a C-terminal Glycine-rich domain [1, 8, 10]. While TDP-43 is predominantly a nuclear protein, in ALS motor neurons it was found to mislocalize to the cytoplasm, with some affected neurons even showing complete clearance of TDP-43 from the nucleus [3]. In addition to this mislocalization, TDP-43 was also found to be modified and to be misfolded in an aggregated state [3]. Its modifications include ubiquitination, phosphorylation, and cleavage [3, 11]. The implications of these modifications as well as their origins and roles in ALS pathology, remain largely unclear and require further investigation [11].

## 3. FUS Is Also an ALS Disease Protein

 The identification of the RBP, TDP-43, as a causative agent of ALS-directed research towards the possibility that other RBPs might also play a role in ALS. This led to the contemporaneous discovery by two groups of the ALS disease protein Fused in Sarcoma, or FUS [4, 5]. Like TDP-43, FUS is a ubiquitously expressed protein that is normally nuclear but is found in cytoplasmic inclusions in ALS brain [4, 5].

FUS contains an N-terminal QGSY-rich domain, followed by a Glycine-rich domain, an RRM, and two RGG-rich domains [1, 10]. Like TDP-43, FUS has roles in a number of different RNA-processing activities, including splicing and mRNA export to the cytoplasm [10]. To date, about 50 ALS-linked FUS mutations have been identified [10].

## 4. TDP-43 and FUS Are Highly Prone to Aggregation and Contain Predicted Prion Domains

 Prions are infectious proteins that are prone to misfold and aggregate and can spread from organism to organism in a DNA/RNA-independent manner [12–17]. They accomplish this by converting natively folded conformers with the same primary sequence into the prion state [14, 15]. Prions cause a number of deadly mammalian diseases, such as bovine spongiform encephalopathy and Creutzfeldt-Jakob disease in humans [12]. A similar process to the spreading of prions might be involved in the cell-to-cell transmission of disease proteins in a number of neurodegenerative diseases, including Alzheimer's disease, Parkinson's disease, and Huntington's disease [13, 16–18].

TDP-43 and FUS both contain a predicted prion domain similar to those found in yeast prion proteins like Sup35 and Ure2 [13, 16, 18–20]. Prion domains confer prion behavior by transitioning from an intrinsically unfolded conformation to a variety of infectious amyloid forms [13–16, 18]. In TDP-43 the predicted prion domain (amino acids 277–414) largely overlaps with the C-terminal Glycine-rich domain, whereas in FUS it resides in the N-terminal QGSY-rich and Glycine-rich domains (amino acids 1–239) [13, 16, 18]. The presence of predicted prion domains and the incorporation of these RBPs into disease-associated inclusions suggested that these RBPs are highly prone to aggregation [13, 16, 18]. It should be noted, however, that neither TDP-43 nor FUS aggregates have been shown to harbor infectious properties, that is, to be self-templating conformers that are naturally transmitted between individuals and promote a self-perpetuating phenotypic change [16]. Indeed, it is not yet clear whether full-length FUS or TDP-43 aggregates are self-templating [16, 21, 22], although specific TDP-43 fragments that harbor the predicted prion domain can access self-templating forms [23]. However, an interesting aspect of ALS is the spreading of pathology to neighboring anatomic regions, particularly in the brain [24]. While this suggests that prion-like behavior is involved in ALS, a more direct demonstration that TDP-43 and FUS can act as bona fide prions is currently lacking.

To test the prediction that these RBPs are intrinsically aggregation-prone, a cell-free system was established to monitor the propensity of pure protein for spontaneous aggregation [21, 22, 25]. Both TDP-43 and FUS were found to rapidly aggregate in vitro, with a lag phase that was either on the order of minutes or too short to measure [21, 22, 25]. Interestingly, both proteins form pore-shaped oligomers and filamentous aggregates that closely resemble the structure of pathological aggregates detected in motor neurons of ALS patients [21, 22, 25].

## 5. Yeast Can Be a Useful System to Model Protein Misfolding Connected with Neurodegeneration

 The baker's yeast *Saccharomyces cerevisiae* has been extensively used to gain insight into many neurodegenerative processes [26]. Furthermore, since disease proteins recapitulate many aspects of pathogenesis in yeast, studying TDP-43 and FUS in yeast was an immediately attractive option [26]. Importantly, overexpression of either TDP-43 or FUS in yeast results in two key aspects also seen in human cases: cytoplasmic aggregation and toxicity [21, 22, 27–34]. This finding suggests that yeast can be used as a model system to study the misfolding and toxicity of ALS proteins.

## 6. ALS-Linked Mutations in TDP-43 and FUS Are Likely to Cause Disease by Distinct Mechanisms

 Though TDP-43 and FUS share many striking similarities [10], several lines of evidence indicate that their respective ALS-linked mutations cause disease by distinct pathways [18]. Many of the TDP-43 ALS-linked mutations localize to the C-terminal Glycine-rich domain [10], which is thought to be critical for TDP-43 misfolding since this is also the location of the predicted prion domain [13, 21, 29]. On the other hand, most of the FUS ALS-linked mutations reside in the extreme C-terminal region, which is also the location of the nuclear localization signal (NLS) of FUS [10]. FUS has a nonclassical NLS comprised of highly conserved proline and tyrosine residues (PY-NLS), which is decoded by the nuclear import factor Transportin-1 (TNPO1) [35].

Most of the TDP-43 ALS-linked mutations tested to date accelerate aggregation in vitro, whereas the FUS mutations have no effect [21, 22]. Q331K and M337V, for instance, are particularly aggressive TDP-43 variants that aggregate more rapidly than WT [21]. The FUS mutants H517Q, R521C, and R521H do not affect the aggregation kinetics of FUS [22]. The presence of the FUS mutations in the PY-NLS suggested that these could disrupt its cellular localization rather than its aggregation [35, 36]. To test this prediction, GFP-tagged WT-FUS as well as C-terminal FUS mutants were expressed in a mammalian cell line and FUS localization was examined [36]. The C-terminal mutations, including R521C, R524S, R522G, and P525L, caused FUS mislocalization to the cytoplasm, in a manner dependent on the severity of the mutations in altering the integrity of the PY-NLS [35, 36]. Interestingly, the degree of cytoplasmic mislocalization was correlated with mean age of disease onset, with mutations (e.g., P525L) causing the greatest cytoplasmic mislocalization also resulting in the earliest age of disease onset [36]. Furthermore, knockdown or inhibition of TNPO1 also resulted in WT-FUS cytoplasmic mislocalization, indicating that the FUS-TNPO1 interaction is crucial for proper cellular localization [36]. Together, these data provide strong evidence that specific ALS-linked TDP-43 mutations primarily cause disease by promoting aggregation, whereas specific ALS-linked FUS mutations cause disease by perturbing nuclear localization [18].

## 7. A Yeast Screen Identified TAF15 as a Putative ALS Gene

 Given the largely overlapping functions and protein domains of TDP-43 and FUS, it is likely that many other RBPs play a crucial role not only in ALS but also in neurodegeneration more generally [16, 18, 25]. This tantalizing possibility led Couthouis et al. to perform a yeast functional screen in an attempt to harness the power of yeast to identify new candidate ALS disease genes [25].

First, the entire human genome was surveyed for proteins that contained RRMs [25]. Of the 213 human genes containing RRMs, Couthouis et al. were able to clone 133 of them into yeast expression vectors as YFP fusion proteins [25]. Next, to narrow the list to those proteins that had similar phenotypes to TDP-43 and FUS in yeast, 38 were identified that were toxic and formed cytoplasmic foci [25]. Of these 38, a bioinformatics approach was taken to rank these in order of strongest to weakest predicted prion domains [25]. 13 of these 38 proteins contain a predicted prion domain and at the top of this list sat TAF15 [25].

Interestingly, TAF15 shares many similarities with TDP-43 and FUS [16]. Its domain architecture is very similar to FUS; it contains an N-terminal QGSY-rich domain, a Glycine-rich domain, an RRM, two RGG domains, and a C-terminal PY-NLS. Like FUS, it contains a predicted prion domain (amino acids 1–152) that overlaps with its QGSY-rich domain [16]. Furthermore, pure TAF15 also aggregates with extremely rapid kinetics and forms aggregates that are morphologically similar to those formed by FUS [25]. Sequencing of the TAF15 gene in ALS and control populations led to the successful identification of several mutations enriched in ALS patients, including M368T, G391E, R408C, G452E, and G473E, which are absent in control populations [25]. Furthermore, most of these mutations cause TAF15 to aggregate more rapidly in vitro and in vivo, similar to TDP-43 ALS-linked mutations [25]. Although these specific TAF15 variants have not been connect to fALS, the search is ongoing [25]. Nonetheless, this study underscores the power of yeast as a model system to study the misfolding of disease-associated proteins and the potentially general role of RBPs in neurodegeneration [25].

## 8. Conclusions

 The identification of TDP-43 as an ALS gene altered the genetic landscape of neurodegenerative disease. The importance of altered RNA processing in disease pathogenesis is now much more widely appreciated. Furthermore, the identification of other RBPs that all contain RRMs and prion domains is unlikely to be mere coincidence [16, 18]. Indeed, it is highly likely that future research will identify many other RBPs and related proteins in neurodegeneration [16]. For instance, recent evidence has linked EWSR1, another RBP very similar to FUS and TAF15, to frontotemporal lobar degeneration with ubiquitin-positive inclusions (FTLD-U) [37]. Additionally, it is likely that the DNA or RNA targets of these RBPs play a significant role in disease [33, 38–41]. Remarkably, a hexanucleotide intronic repeat was recently identified that can account for a large fraction of ALS cases [42, 43]. Further examination and elucidation of these processes involving RBPs in disease will be critical.

Though much progress has been made in the identification and characterization of the roles that RBPs play in ALS, much is still beyond our current understanding. For instance, it is still not known whether these proteins cause disease by a loss of function or gain of function or both [1, 10, 11, 16–18]. It is still unclear how certain ALS-linked mutations cause disease. For example, some TDP-43 ALS-linked mutations do not promote aggregation [21] and several FUS ALS-linked mutations lie outside of the PY-NLS [10]. It is unclear whether these mutations cause FUS to aggregate more rapidly, to interact with other proteins, or to disrupt its normal RNA targets. The role of specific RNA targets with which these RBPs interact may also be key to disease pathogenesis [38–41]. Ultimately, an enhanced understanding of the exact pathways these RBPs take en route to disease onset is required to truly understand pathogenesis and to develop effective therapeutic remedies.
